# Adherence to e-health interventions for substance use and the factors influencing it: Systematic Review, meta-analysis, and meta-regression

**DOI:** 10.1177/20552076231203876

**Published:** 2023-09-28

**Authors:** Farhud Shams, Andy M.Y. Tai, Jane Kim, Marisha Boyd, Maximilian Meyer, Alireza Kazemi, Reinhard Michael Krausz

**Affiliations:** 1 Department of Psychiatry, 12358 Faculty of Medicine, University of British Columbia, Vancouver, BC, Canada; 2 Department of Psychiatry, University of Basel Psychiatric Clinics, University of Basel, Basel, Switzerland

**Keywords:** adherence, e-Health interventions, substance use disorder, systematic review, meta-Analysis, meta-Regression

## Abstract

**Background:**

Substance use disorders affect 36 million people globally, but only a small proportion of them receive the necessary treatment. E-health interventions have been developed to address this issue by improving access to substance use treatment. However, concerns about participant engagement and adherence to these interventions remain. This review aimed to evaluate adherence to e-health interventions targeting substance use and identify hypothesized predictors of adherence.

**Methods:**

A systematic review of literature published between 2009 and 2020 was conducted, and data on adherence measures and hypothesized predictors were extracted. Meta-analysis and meta-regression were used to analyze the data. The two adherence measures were (a) the mean proportion of modules completed across the intervention groups and (b) the proportion of participants that completed all modules. Four meta-regression models assessed each covariate including guidance, blended treatment, intervention duration and recruitment strategy.

**Results:**

The overall pooled adherence rate was 0.60 (95%-CI: 0.52–0.67) for the mean proportion of modules completed across 30 intervention arms and 0.47 (95%-CI: 0.35–0.59) for the proportion of participants that completed all modules across 9 intervention arms. Guidance, blended treatment, and recruitment were significant predictors of adherence, while treatment duration was not.

**Conclusion:**

The study suggests that more research is needed to identify predictors of adherence, in order to determine specific aspects that contribute to better exposure to intervention content. Reporting adherence and predictors in future studies can lead to improved meta-analyses and the development of more engaging interventions. Identifying predictors can aid in designing effective interventions for substance use disorders, with important implications for e-health interventions targeting substance use.

## Introduction

An estimated 269 million individuals worldwide used drugs at least once in 2018, which represents a 30% increase from 2009.^1^ In 2022, around 284 million people aged 15–64 used drugs worldwide, a 26 % increase over the previous decade.^2^ Considering the increase in the global population, it is projected that by 2030, the number of individual drug users worldwide will rise by 11%, amounting to 299 million.^3^ According to estimates, the worldwide prevalence of substance-related disorders has led to a considerable burden, with a total of 494,000 fatalities attributed to illicit drug use in 2019,^4^ reflecting a 5% increase between 2017 and 2019.^4^ Among these deaths, 128,000 were directly related to drug use, with most of them being attributed to overdoses. In contrast, 366,000 deaths were due to indirect reasons, including liver cancer, HIV and AIDS, self-harm associated with drug use and others.^4^ In 2019, substance use disorders (SUDs) were projected to account for a global loss of 21 million disability-adjusted life years annually.^4^

Compared to the rest of the world, North America has been experiencing an overdose crisis, with nearly 45,000 individuals died from an opioid overdose in the United States in 2018 and over 4000 opioid-related deaths occurred in Canada in the same year.^5^ This number increased to 107,622 in the United States in 2020 and 7993 in Canada in the same year.^6,7^ One major challenge of the system of care is the availability of resources, which is limited concerning the incidence, prevalence, and distribution of substance use (SU) conditions.^8^ Many also experience barriers in accessing treatment due to discrimination and stigma, particularly those in correctional settings, ethnic minorities, immigrants, and refugees.^3^ Globally, in the period between 2015 and 2019 approximately 36 million individuals had an SUD; however, only 7 million people, or approximately 19% of those in need received treatment.^2,3^

Virtual care is a significant field that has attracted the attention of mental health care researchers and clinicians for its ability to address the gap between needs and resources. ‘E-health’ is an umbrella term that refers to the use of technology to address health-related issues, such as web-based interventions for mental health and SU. These interventions refer to systematic approaches or programs designed to bring about desired changes or improvements in a person's health status or well-being. Sometimes, the more specific term ‘e-mental health’ refers to e-health interventions that target people seeking help for mental health and SU problems.^9,10^ Web-based interventions have proven effective for various mental health disorders including depression, panic, post-traumatic stress disorder, stress, insomnia, and eating disorders.^11^ Web- and mobile-based interventions have also been used to target a range of SU behaviours and have demonstrated effectiveness in previous meta-analyses.^12–20^ Web-based interventions for SU have demonstrated small to moderate effects in previous meta-analyses.^12,13,17–19^ Boumparis et al.^12^ concluded that internet interventions for adult illicit substance users demonstrated small but significant effects across various populations. These online interventions are often delivered through modules, which refers to distinct units or components within an intervention program. It is a self-contained unit that focuses on a specific aspect of the overall intervention. Modules are often designed to be delivered sequentially or in a structured manner to address different aspects of a health condition or to achieve specific objectives. Each module typically has its own set of goals, content, and activities.

Measuring the effectiveness of e-health interventions involves evaluating their impact on various outcomes, such as health behaviors, clinical outcomes, patient satisfaction, or cost-effectiveness. The measurement this study seeks to investigate is the extent to which participants or users comply with or follow the recommended or prescribed behaviors, activities, or treatment protocols within the e-health intervention. While there are many similar terms, adherence has been most often used as a term to try and describe “true program usage”. Adherence is more accurate than study attrition in terms of describing the actual exposure to the intervention (since some people might drop out because they were successful in their treatment) and can be more easily and objectively measured than engagement. For the purposes of this review, we defined adherence as the “extent to which individuals experience the content of an intervention”.^11,21^ To operationalize this definition in an outcome measure that helps us understand the degree of adherence as best as possible, we set out to explore the following two measures: (a) the number of modules completed and (b) the percentage of participants completing all modules. The number of modules completed has previously been positively associated with better outcomes.^22^

A few studies have been published on the predictors of adherence in digital interventions.^22–24^ One systematic review, which looked at the impact of adherence on the effectiveness of e-therapies, reported better outcomes in depression and anxiety scores associated with higher levels of adherence as measured by the number of modules completed.^22^ However, no associations were found when adherence was measured in terms of number of logins, self-reported activities, time online and pages opened.^22^ The same review also mentioned several limitations when it came to studying adherence. They reported that out of 69 studies, only 33 examined the relationship between adherence and outcomes. In addition, adherence is not clearly defined, and adherence measures varied considerably across different studies. This made it infeasible to conduct a meta-analysis and challenging to draw firm conclusions in the narrative synthesis.^22^ Other reviews have been conducted on adherence in interventions targeting sleep disorders.^25,26^ Reviews of adherence either investigated the relationship between adherence and primary outcomes, the predictors of adherence in that domain or both.

The first hypothesis states that any form of guidance, whether from a clinician or a non-clinician, will predict greater adherence than receiving no guidance, while clinician versus non-clinician guidance will result in similar levels of adherence. Unguided e-health interventions, also described as self-help e-health interventions, are fully automated interventions that participants can complete without human guidance. Guided interventions, on the other hand, provide some sort of human support to guide participants through the intervention content, for example through phone calls, reminder messages or in-person contact. The guide or supporter may be a professional (such as a clinical psychologist or psychiatrist), a trained mental health worker or a graduate student (non-clinician). The role of the guides may solely be to remind participants to use and engage with the intervention material, which is a common strategy employed in non-clinician guidance. Technical support such as an introduction on how to use the system will not be considered as guidance for the purpose of this review. There is evidence in the literature that suggests that guidance may lead to better effect sizes than no guidance. A review of systematic reviews and meta-analyses in the literature on internet-based interventions for the treatment of mental health supports the premise that guided interventions seem to be more effective than unguided interventions and are comparable in effectiveness to face-to-face interventions, at least for anxiety and depression.^27,28^ An individual patient data meta-analysis of alcohol internet interventions reported that guided interventions were significantly more effective compared to unguided or self-help e-health interventions.^29^ A review of reviews noted that generally, larger effects were observed when interventions included some form of guidance, feedback, or reminders.^30^ Meanwhile, a review of e-health cannabis interventions in 2013 did not find a difference in effect sizes between guided and unguided (self-help) type interventions.^31^ In accordance with a suggestion from Tait et al.,^31^ we speculate that “those with more severe problems would benefit more from additional therapist assistance”, which implies that there may be other factors correlated with guidance when it comes to predicting adherence and/or treatment effects, such as the severity of use and duration of SU. Unfortunately, our preliminary review revealed large heterogeneity in the reporting of these participant characteristics in e-health studies, and in combination with the limitations of patient characteristics in meta-regressions, it would not be feasible to model these factors. Most of the mentioned reviews described the possible association between guidance and effect sizes, but not the relationship between guidance and adherence, although increasing adherence to the content has been described as one of the main goals of providing guidance in e-health interventions.^32,33^ To address this gap and assess if guidance predicts adherence outcomes in studies of e-health interventions for SU, we included both a categorical predictor (clinician-guided, non-clinician-guided, and unguided) and a dichotomous predictor (guided and unguided).

Second, it was hypothesized that blended treatment would result in greater adherence. Blended interventions pair face-to-face conventional treatments (such as individual or group therapy) with e-health interventions. This integration of e-health into treatment is intended to improve the therapeutic process (e.g. adherence) or increase treatment efficacy and effectiveness.^34–37^ It has been suggested that pairing interventions may lead to better results in terms of engagement and adherence;^28^ thus we included blended treatment as a dichotomous predictor variable for adherence.

Third, it was hypothesized that interventions with shorter durations (measured as the number of weeks in treatment) would result in higher adherence rates compared to interventions with longer durations. Some studies have noted that engagement with e-health websites tends to drop quickly after the initial weeks of participation.^38,39^ One review of adherence to web-based interventions looked at intervention duration as a predictor of adherence in different domains of health (including mental health), but did not find any significant effects on adherence.^24^ The literature on intervention duration and related measures and their effects on adherence is limited. For this reason, the present review sought to examine whether there is an association between intervention duration and adherence.

Fourth, it was also hypothesized that a local recruitment strategy would result in higher adherence than a global recruitment strategy. Local recruitment includes recruitment that was conducted at local care settings (e.g. treatment centers, hospitals, etc.), while global recruitment strategies use widespread media (e.g. TV ads, social media, and the web) to recruit participants. Recruitment that was conducted through the web and included promotional links on websites of local treatment centers would be considered global (for example see Schaub et al.^40^). Local recruitment may inherently involve additional accountability, for example through direct physician referrals and more personal contact (in-person visits or calls during the recruitment process). Since global recruitment strategies through the web are a relatively recent method that was introduced with e-health, we wanted to understand their possible effects on adherence outcomes.

In expanding upon the current literature, this review aims to highlight some important factors that may predict adherence. The objectives were to (a) summarize existing rates of adherence in studies of e-health interventions for SU using two measures and conducting two separate meta-analyses, and (b) examine the impact of four possible predictors of adherence through meta-regression.

## Methods

### Data sources and search strategy

A literature search was conducted on 19 February 2021. The databases searched included Medline (Ovid interface), EMBASE (Ovid interface) and PsycInfo (EBSCO interface). Searches were limited to publication language (English) and year (2009–present). The search strategy consisted of three groups of key concepts: (a) substance use– and addiction-related keywords, (b) e-health- and technology-related keywords, and (c) the keywords intervention, and treatment. Keywords under each group were combined with the “OR” operator, while the groups were merged with the “AND” operator. The three groups helped in limiting results to find papers that focused on interventions or treatments for SU using web-, computer-, or mobile-based components. Adherence-related keywords were not included in the search terms to allow for a broader search scope and reduce the risk of missing studies that did not focus on adherence. Detailed search terms can be seen in Appendix A. The Covidence tool was utilized throughout the selection and data extraction process. This review did not publish a study protocol. However, the authors adhered to an a priori methodology, and the methods were not altered after the screening process.

### Study selection criteria/eligibility criteria

Eligible studies included those of e-health interventions targeting SU in adult populations. Samples with a mean age of 18 or less were excluded. Multiple types of study designs were included such as randomized controlled trials (RCTs), quasi-experimental and pilot/eligibility study designs. Since the main outcome of interest was a process outcome rather than a measure of effect size, our selection criteria also set out to include observational studies. However, only one observational study met the selection criteria and was included in the review. Another criterion for inclusion was that the intervention was designed to be used across multiple sessions, which led to the exclusion of single-session interventions such as brief interventions (e.g. Arnaud et al.^41^) and personalized normative feedback interventions such as E-CHUG (e.g. Doumas et al.^42^). These were excluded because the primary measure for adherence is the number of modules that were completed over several sessions. For studies to be included, they had to report data related to adherence, and dropout/retention and adherence had to be distinguishable. Studies also had to provide modules as part of a regular schedule over multiple sessions. Interventions that were aimed at care providers or relatives of the substance user were excluded. We also excluded prevention programs for the following reasons: (1) Most prevention programs were targeted at adolescents under the age of 18. (2) Most prevention programs were aimed at family or relatives of the participant. (3) Prevention aims at a different target population than treatments. Additional study selection criteria included (a) published in English; (b) published after 2009; (c) a trial of SU intervention; (d) not targeting only caffeine or nicotine; (e) article is not a review, development paper or opinion paper with no primary data reported; and (f) the study includes at least one of the two measures of adherence used for the meta-analysis. The two measures were as follows: (a) the mean number of modules completed and (b) the proportion of participants who completed all modules.

### Study selection

Studies were assessed for eligibility in two phases: screening and full-text review. In the screening stage, titles and abstracts were reviewed for preliminary inclusion. Screening was conducted by two reviewers with only one vote required for inclusion. To assess the reliability of the decisions, and to develop a process and refine the selection criteria, the two reviewers AT and MB, first conducted a title and abstract pilot screening for a subsample of identified articles (n = 200). After this pilot screening, the selection criteria did not change. After screening through all the identified articles, eligibility was further determined through full-text reviews by the two reviewers. During this stage, the primary target was to identify whether the studies reported any data on adherence, since most other criteria were mentioned in the title or abstract (such as study design, target population, and the type of intervention). Nonetheless, all criteria were double checked in the full-text review stage. In both stages of reviews, whenever consensus could not be reached, a third reviewer (FS) helped resolve any uncertainties through discussion. FS also screened a subsample of the excluded references (n = 600) to make sure that important studies meeting the inclusion criteria were not missed.

### Data extraction

For each included study, the reviewers recorded study identifiers: name of the study, publication year and author list. Additionally, the characteristics of the studies were recorded. These included information such as the country where the study was conducted, study design, and method of recruitment. Intervention characteristics that were recorded were the type of intervention (e.g. cognitive behavioural therapy (CBT) and motivational interviewing (MI)), mode of delivery (web vs offline), methods of recruitment, target of the intervention (general SU or specific substances), details of the guidance provided, whether the treatment was blended (e.g. an e-health component provided as an adjunct to existing conventional treatment) and the duration of the treatment. For the meta-analysis of adherence, for each relevant intervention arm of studies that met the inclusion criteria, we extracted the mean number of modules completed, the standard deviation of that mean, the total number of modules provided (in some studies, only the core modules were included) and the number of participants for whom these data were reported (e.g. the number of participants who started using the program).

### Analysis

For each intervention arm, the proportion of completed mean modules and the standard deviation of this mean were calculated by dividing the mean and standard deviation of the completed modules by the total number of modules. In cases where the intervention clearly distinguished between core and optional modules, only the core modules were used as the total to calculate the percentage of completed mean modules, since the participants are less likely to complete modules beyond the core modules. For the secondary measure, if the proportion of participants that completed all modules was reported as a count of participants, we calculated the percentage by dividing it through the total number of participants. Standard deviations were calculated manually if a study reported the standard error of the mean instead. Descriptions of the authors regarding recruitment strategies were used to categorize studies into global or local recruitment. For each intervention or study arm, author descriptions were used to identify whether the intervention was clinician-guided, non-clinician-guided or unguided, as well as to identify whether the intervention arm used an integrated blended treatment model.

All analyses were conducted using RStudio (version 1.3.1093) and R (version 4.0.3). The R packages used were meta, dmetar and metafor. Two meta-analyses were conducted for two different types of adherence measures: (a) the mean proportion of total modules completed by participants and (b) the proportion of participants who completed all modules. Authors of the included studies were contacted for data when adherence was reported, but some data were missing. All participants who had completed pre-treatment or baseline assessments and had been allocated to an e-health intervention arm were included in the analyses, regardless of whether the participant started the intervention.

For the primary meta-analysis, heterogeneity was calculated using the *I^2^* index, expressed in percentages. It provides an estimate of the variability that is due to true variations in outcomes (as opposed to variation due to sampling error). The Q-statistic was used to test whether the heterogeneity was statistically significant. High heterogeneity was expected due to differences among studies and populations, which is why a random effects model was used for the meta-analysis of the means. The random effects model expects not only for random error within the studies, but also for between-study variation due to true differences. The random effects model has also been recommended for meta-analyses regardless of the degree of heterogeneity.^43^ The restricted maximum likelihood estimator was used to calculate the heterogeneity variance.^44^ We used Knapp–Hartung adjustments to calculate the confidence intervals around the pooled mean.^45^ The secondary meta-analysis also employed a random effects model with the same statistics reported. The percentage of participants who completed all modules was used in a meta-analysis of prevalence. Some studies also reported the proportion of participants who completed a certain proportion of modules (e.g. 80%) and some reported the number of completers for each module.

Next, meta-regressions were used to understand whether hypothesized predictors had an effect on adherence. For each predictor, a random-effects meta-regression model was constructed. It has been suggested that misleading results may be produced when using fixed-effects meta-regressions.^46^ The recommended minimum number of observations needed for each covariate is 10.^47,48^ For the same reason, multiple meta-regressions have not been used in the present review, as they are not recommended when the number of studies is small, as the ratio of studies to covariates must be appropriately large.^48^ Aggregation bias is an issue in meta-regressions because summarized patient characteristics (e.g. average age, gender, average duration of follow-up) do not necessarily reflect the individual patient characteristics within the study.^49^ This may lead to an ecological fallacy because of the mismatch in the unit of analysis. Relationships across studies may not reflect relationships within studies. These relationships are best measured within a study. In order to avoid this bias, since, in contrast to an individual patient data meta-analysis, we were not able to separate within-study and between-study variance for participant-level characteristics, we decided to only include study- and intervention-level characteristics as predictors of adherence.

## Results

### Study selection

**Figure 1.**
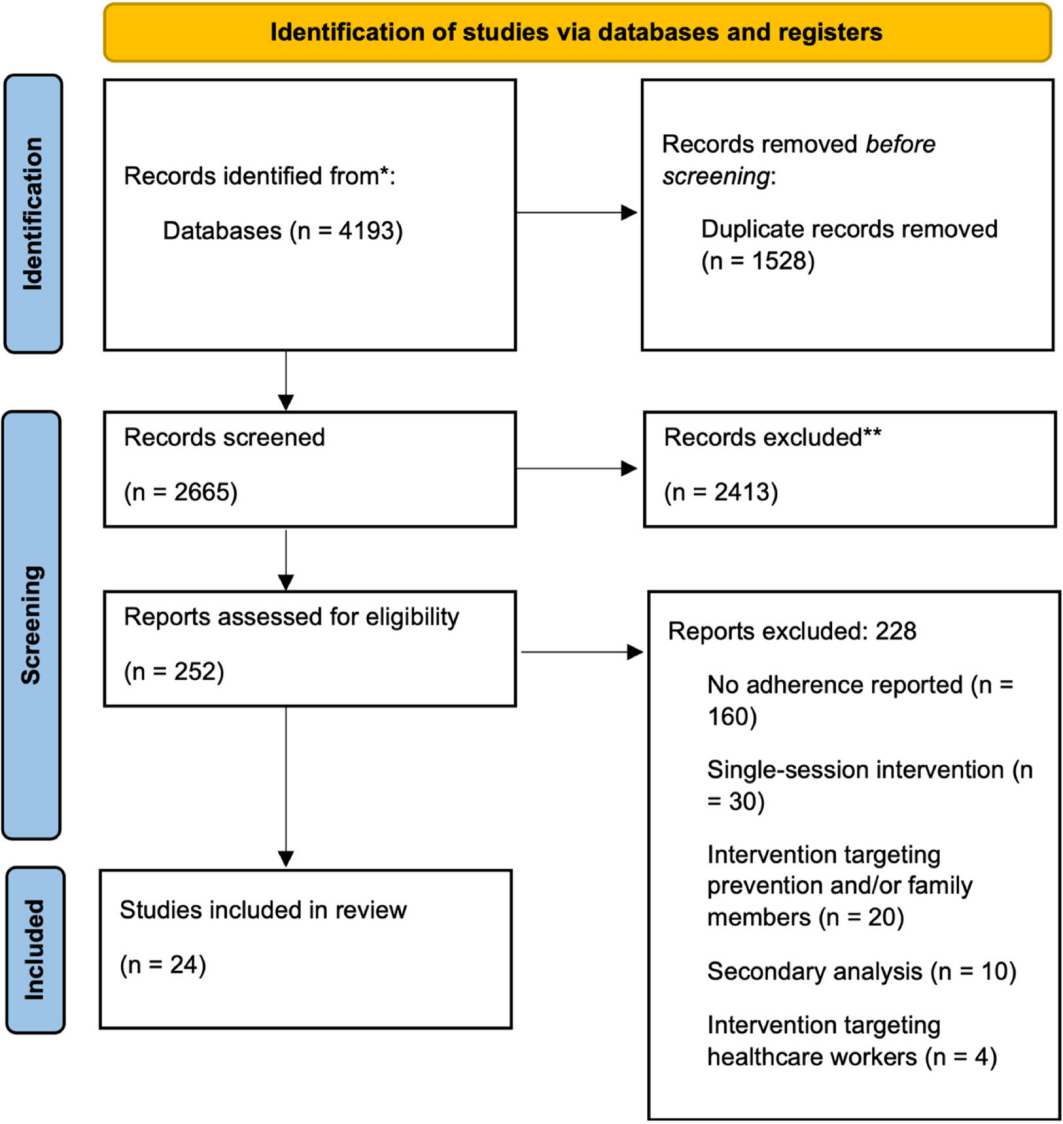
Selection process of studies for inclusion in the present review and analyses.

We identified 4193 records from three databases. After removing 1528 duplicates, 2665 records were screened for inclusion based on titles and abstracts, of which 2413 were excluded. The remaining 252 records underwent a full-text review, which led to the final number of 24 included studies that met the inclusion criteria and were selected for our meta-analyses. Additional data related to adherence had to be requested from the authors of five studies, of which four responded and provided the necessary data points that were missing. One author group did not respond and was excluded from the analyses at the full-text review stage. A PRISMA flow chart is illustrated in Figure 1.

### Analysis

#### Overview of studies

Twenty-four studies published between 2008 and 2020 were included (for details, see Table 1). Many studies originated in the United States (n = 13). Other countries included Australia (n = 3), Sweden (n = 3), Switzerland (n = 3), Japan (n = 1) and Mexico (n = 1). Most studies employed a randomized controlled design (n = 18) with some others providing variations of that design, such as pilot designs or open-label non-blinded designs. One study also used a non-randomized experimental trial design. Fifteen of the 24 studies recruited their participants through a single or multiple local treatment centers or clinics. Only four studies used a recruitment process that was fully online and remote. Regarding intervention targets, nine studies targeted general substance/alcohol use and eight studies targeted alcohol and/or cannabis use. Other substances that were targeted included cocaine (n = 4), opioids (n = 2) and stimulants (n = 1). Additional information can be found in Appendix A.

**Table 1.** Meta-analysis data of included studies.

Study ID	N	# Modules	Mean	SD	Completers	% of completers
Acosta 2017 ^50^	81	12	8.8	6.2	31	38.30
Batterham 2018a ^51^	66	10	4	3.7	-	-
Batterham 2018b ^51^	62	10	3.6	3.4	-	-
Brooks 2010 ^52^	14	48	40	11.9	-	-
Budney 2011 ^53^	16	9	6.9	2.8	-	-
Campbell 2014 ^54^	255	48	36.6	18.1	-	-
Carroll 2008 ^55^	35	6	4.3	2.4	-	-
Carroll 2014 ^56^	44	7	5.1	2.3	23	52
Carroll 2018a ^57^	28	7	4.43	2.87	-	-
Carroll 2018b ^57^	38	7	4.95	2.31	-	-
Chaple 2014 ^58^	249	48	34.84	18.71	139	55.8
Deady 2016 ^59^	60	4	1.5	1.53	-	-
Johansson 2017 ^60^	3897	7	2.2	2.2	624	16
Johansson 2020 ^61^	104	5	3.74	1.56	-	-
Kay-Lambkin 2009 ^62^	32	10	7.61	2.87	17	52
Kiluk 2016a ^63^	20	7	5.6	1.9	10	50
Kiluk 2016b ^63^	24	7	5.4	1.9	10	41.7
Kiluk 2018 ^64^	38	7	5.5	2.3	-	-
Marsch 2014 ^65^	80	67	27.56	24.44	-	-
Paris 2018 ^66^	43	7	5.3	2.3	24	56
Schaub 2012 ^67^	18	8	2.6	2.04	-	-
Schaub 2015a ^68^	106	9	2.896	2.777	-	-
Schaub 2015b ^68^	93	9	2.656	2.487	-	-
Schaub 2019a ^40^	114	9	3.43	2.8	-	-
Schaub 2019b ^40^	108	9	2.71	2.44	-	-
Shi 2019 ^69^	10	8	4.2	2	-	-
Sundstrom 2020a ^70^	72	13	8.4	3.9	-	-
Sundstrom 2020b ^70^	71	9	5.9	2.8	-	-
Takano 2020 ^71^	23	6	5.478	1.08	17	73
Tiburcio 2018 ^72^	9	8	4.666	2.179	-	-

Out of the 24 included studies, 12 (50%) were based on CBT, while another 6 (25%) used a combined approach of CBT blended with other known types of treatments such as MI, motivational enhancement therapy (MET) and contingency management (CM). Other approaches included relapse prevention, the community reinforcement approach, behavioral self-management and problem-solving

#### Meta-analysis of the mean proportion of the completed modules

**Figure 2.**
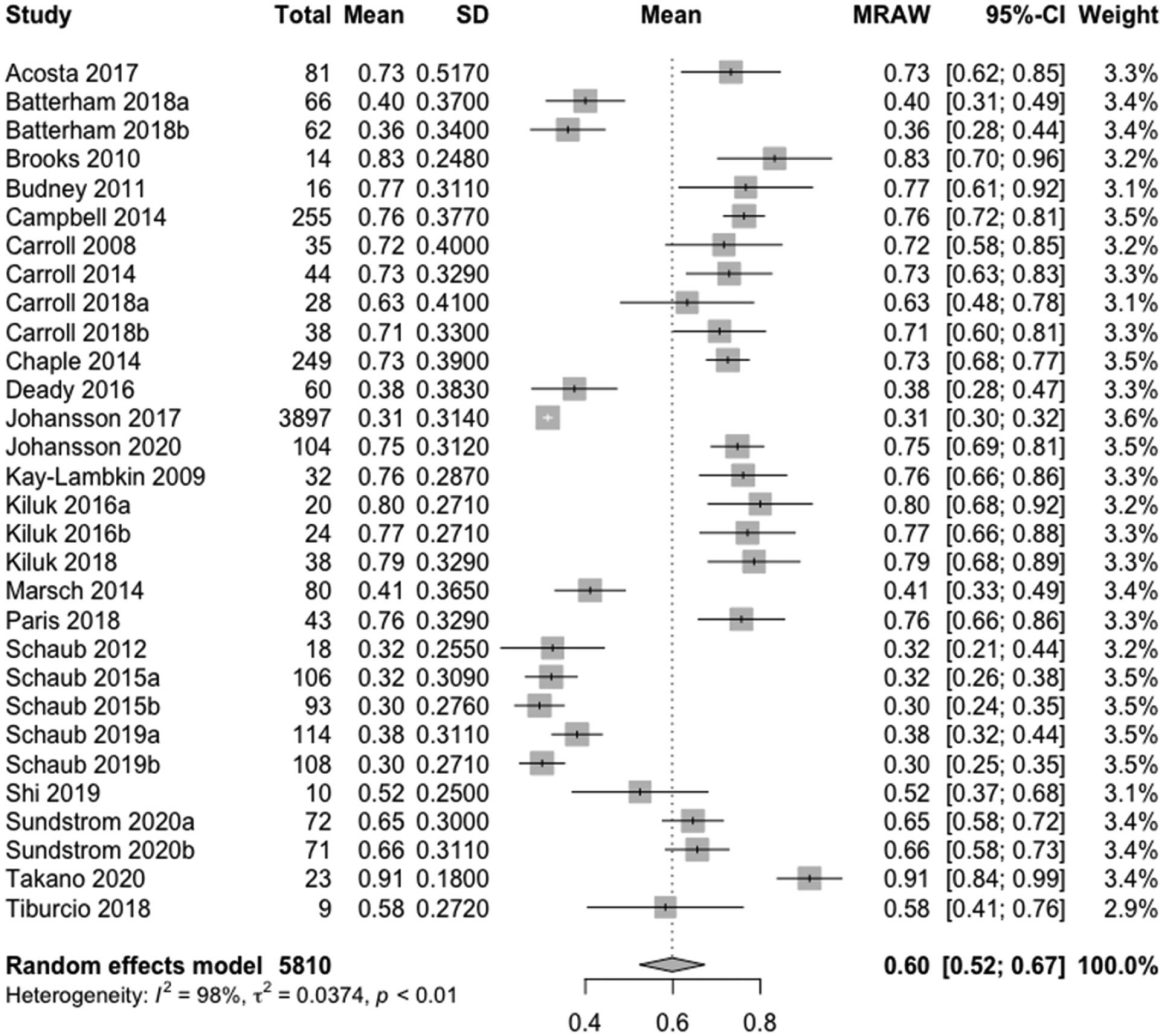
Meta-analysis of the percentage of completed modules: the meta-analysis displays the percentage of mean completed modules, calculated by dividing the mean completed sessions (with corresponding standard deviation) by the total number of sessions. The study column represents the studies included in this meta-analysis. Total indicates the sample size or the total number of participants. Mean represents the percentage of mean modules completed. SD represents the standard deviation of the mean. MRAW represents the mean modules completed based on completed sessions. The 95% confidence interval (95%-CI) is displayed. Weight indicates the assigned proportion based on the contribution to the pooled estimate.

Relevant meta-analysis data were available for 24 studies, of which all reported the mean number of the completed modules. Six studies reported adherence data for two relevant intervention arms, which resulted in a total of 30 rows of data. The pooled mean proportion of modules completed was 0.60 (95%-CI: 0.52–0.67), which means that across the 30 e-health intervention arms, participants completed a mean of 60% of modules. Heterogeneity was considerable (I^2 ^= 98%, p < 0.01), which means that 98% of the observed effect is due to variation in the true effects rather than sampling error (see Figure 2).

**Figure 3.**
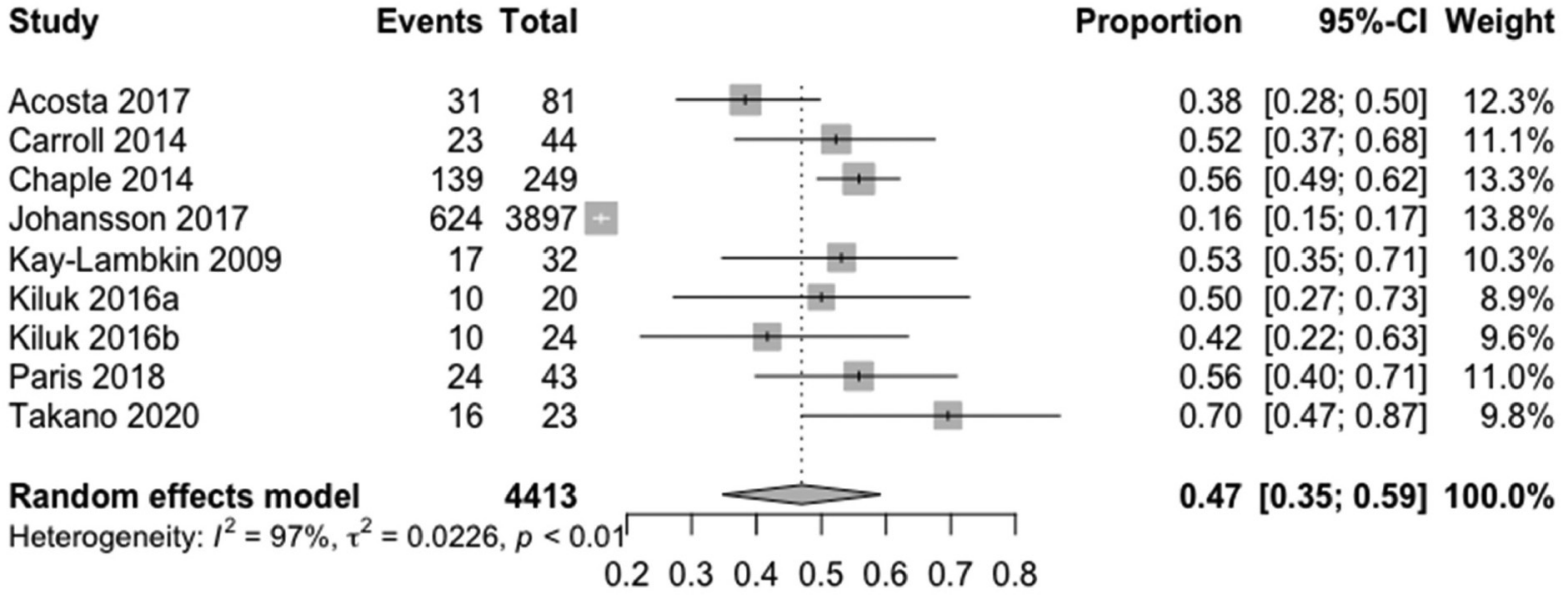
Meta-analysis of the percentage of participants completing all modules: the meta-analysis presents the percentage of participants who completed all modules. The Study column represents the studies included in this meta-analysis. Events indicates the number of participants who completed all modules. Total indicates the sample size or the total number of participants. Proportion represents the study events divided by the total number of events included in this analysis. The 95% confidence interval (95%-CI) is provided. Weight indicates the assigned proportion based on the contribution to the pooled estimate.

Nine studies reported this measure and were included in the secondary meta-analysis. The pooled proportion of participants who completed all modules was 0.47 (95%-CI: 0.35–0.59) with considerable heterogeneity (I^2 ^= 97%, p < 0.01). A forest plot of the meta-analysis is presented in Figure 3.

### Meta-regression

#### Overview

**Figure 4.**
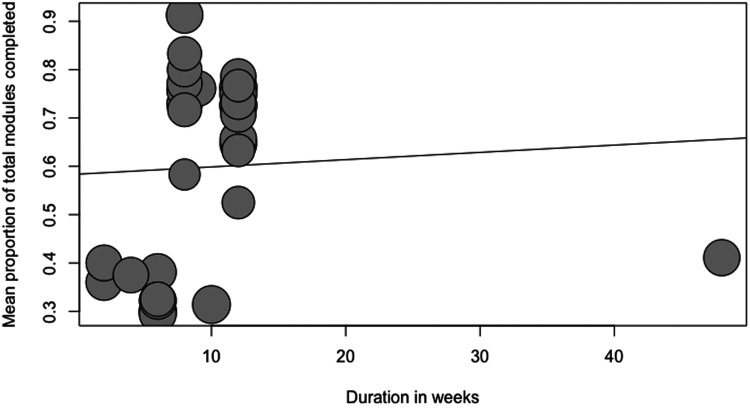
Scatterplot showing the relationship between adherence and duration (in weeks).

**Figure 5.**
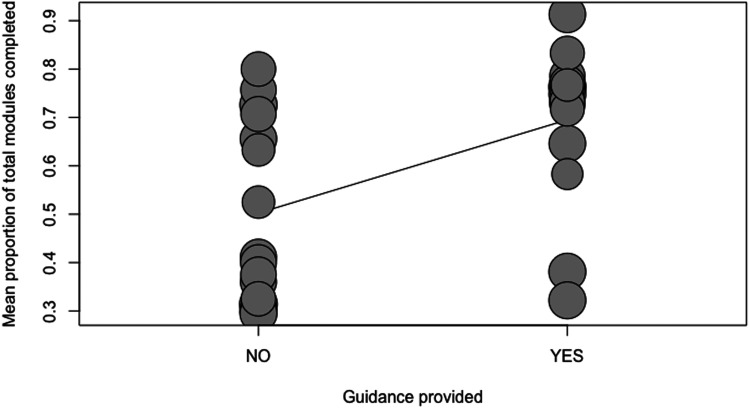
Scatterplot showing the relationship between adherence and guidance.

**Figure 6.**
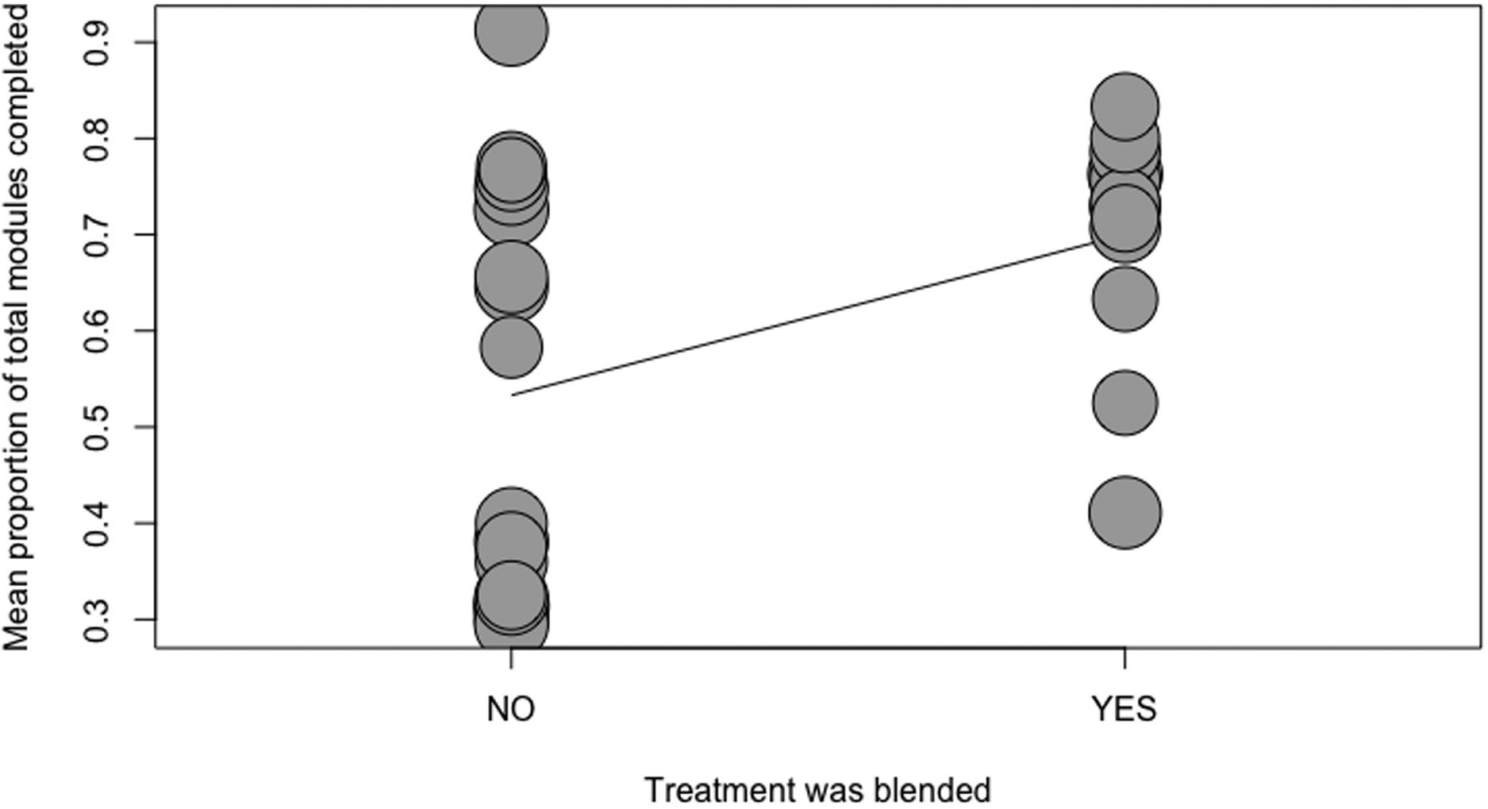
Scatterplot showing the relationship between adherence and blended treatment.

**Figure 7.**
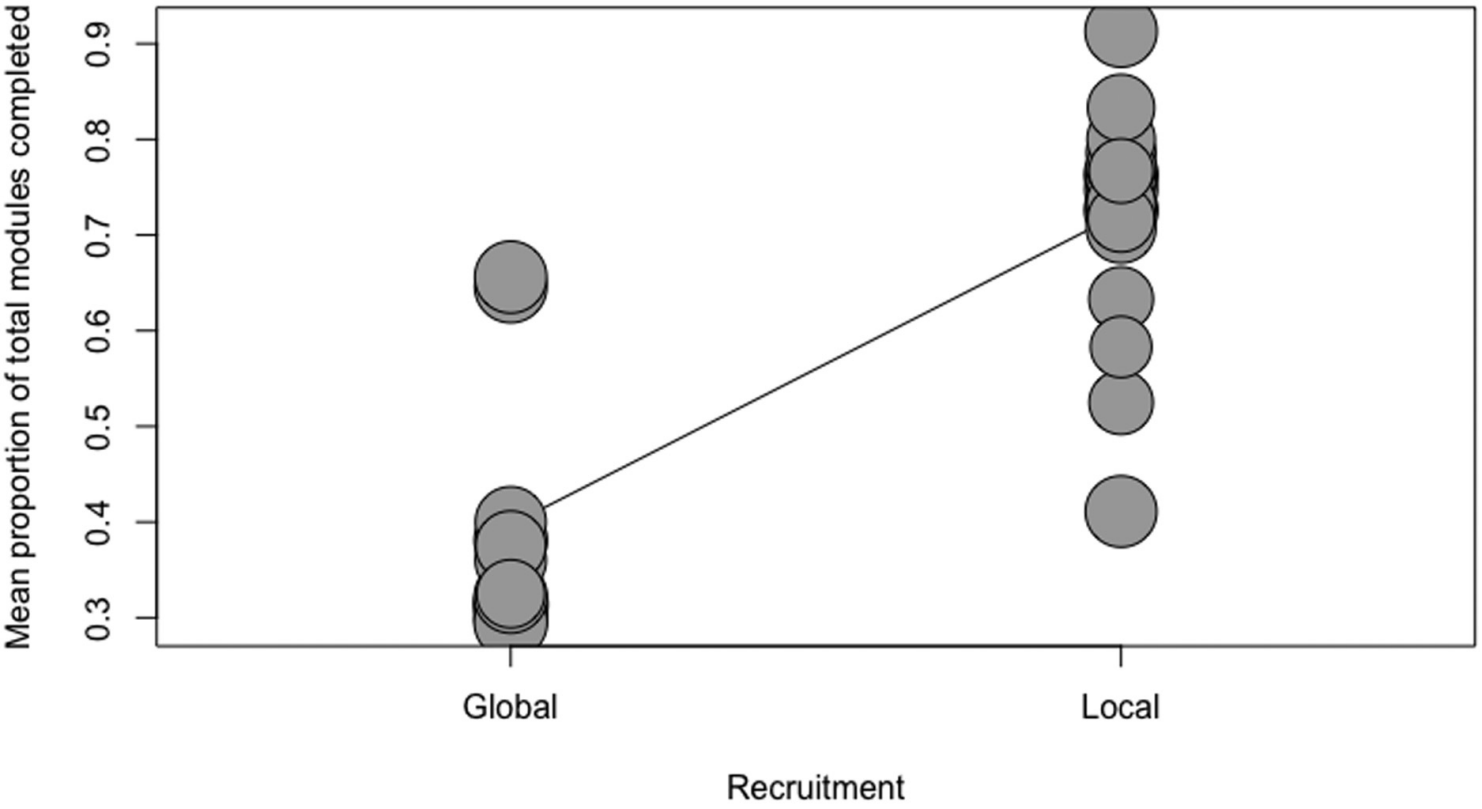
Scatterplot showing the relationship between adherence and type of recruitment.

Four separate meta-regressions were conducted to test the a priori hypotheses. Table 2 represents the number of observations for categorical variables used in the meta-regressions. The results of the four meta-regression models are presented in Table 3. Guidance was a significant predictor of adherence (p = 0.0056; R^2 = 22.19%). On average, adding guidance increased the module completion rate by 19% (ß = 0.19). Only a low number of studies reported the provision of non-clinician guidance (n = 5); thus, a categorical meta-regression comparing clinician versus non-clinician guidance versus no guidance was not feasible. Blended treatment was a significant predictor of adherence (p = 0.0215; R^2 = 15.2%; ß = 0.17). The duration of the intervention (in weeks) was not a significant predictor of adherence (p = 0.7529; R^2 = 0%; ß = 0.002). A local recruitment strategy was a significant predictor of adherence (p < 0.0001; R^2 = 66.6%; ß = 0.33). These relationships have been visualized in scatterplots (see Figures 4–7).

**Table 2.** The number of observations for categorical variables used for meta-regressions.

Variable		Number of studies included
Guidance (dichotomous)		
	Yes	15
	No	15
Guidance (categorical)		
	Clinician	10
	Non-clinician	5
	Unguided	15
Blended		
	Yes	12
	No	18
Recruitment		
	Global	11
	Local	19

**Table 3.** Results of meta-regression models for predictor variables.

Predictor		Model			Predictors		
		F	p	R^2^	B	SE	p
Guidance		9.02	.006	0.22			
	Constant				0.5	0.05	<.0001
	Guidance: yes				0.19	0.06	0.006
Blended treatment		5.93	0.02	0.15			
	Constant				0.53	0.04	<.0001
	Blended: yes				0.17	0.07	0.02
Recruitment		52.5	.0001	0.67			
	Constant				0.4	0.04	<.0001
	Local				0.33	0.05	<.0001
Intervention duration		0.1	0.75	0.00			
	Constant				0.58	0.06	<.0001
	Duration				0.002	0.005	0.75

## Discussion

### Key findings

The study was conducted to show the importance of adherence in effective e-health interventions targeting substance use (SU) and to identify factors that could predict adherence to these interventions. The systematic review involved an extensive search of relevant databases and a rigorous screening process to identify eligible studies that met the inclusion criteria. A meta-analysis was then performed to synthesize the findings from the selected studies and provide a comprehensive overview of adherence in e-health interventions targeting SU.

The four hypothesized factors – provision of guidance, blended treatment model, duration of intervention, and recruitment strategy – were selected based on previous research and expert opinions. The meta-regression analysis allowed researchers to examine the relationship between these factors and adherence, while controlling for other potential confounding variables.

The results of the study provide valuable insights into the factors that can enhance adherence to e-health interventions targeting SU, which can inform the development and implementation of more effective interventions in the future. The study also highlights the need for more rigorous research on this topic, as there is still much to learn about the factors that influence adherence to e-health interventions targeting SU.

### Meta-analysis

The pooled proportion of mean modules completed across studies was 0.60 (95%-CI: 0.52–0.67). Since this was a single-group meta-analysis, there are no comparison groups to compare the adherence to. Unfortunately, since no previous meta-analyses have been conducted on the proportion of mean modules completed in e-health interventions for SU, it is not possible to give weight to this value. Overall, looking at the proportion of mean modules completed within individual studies, where multiple studies have over 70% with the highest being at 91%, there seems to be consequential room for improvement. This can be compared to traditional psychotherapy where residential programs reported a 65% completion rate compared to 52% for outpatient settings.^73^

The proportion of participants who completed all modules is a measure that has been used in previous meta-analyses of adherence,^24^ but it may not be an ideal measure to understand adherence due to the different designs and goals of interventions. Some e-health interventions may have been designed to be exploratory, meaning that participants are presented with a larger number of modules and is not expected to complete all of them. This limitation also partially applies to the other adherence measure, namely the proportion of mean modules completed. To mitigate this issue in future research, studies should consistently report the recommended number of modules participants were expected to complete. For example, some studies separated core modules from optional ones. As described in the Methods section, in such cases, the total number of modules used to calculate the adherence rate included only the number of core modules. This may introduce a limitation due to the possibility of incomplete descriptions of how or if this information was communicated to participants.

For reliable analyses, a careful distinction had to be made between studies that reported mean completed modules versus studies that reported mean modules attended or started. Unfortunately, authors did not always clearly describe how these adherence outcomes were measured. Another issue that was identified relates to the number of participants considered in respect to adherence outcomes reported because it is not always clear if baseline participants quit before they even attempted the first module or session, although some studies specifically stated the number of participants that started the first module or session. This type of reporting had its advantages, since we were looking to understand the actual usage of the participants who attended the intervention and not just of participants who stayed in the study until the follow-up assessments and did not access any content. Unfortunately, some studies only reported on those participants that completed follow-up, thus missing data on participants who dropped out during the intervention. For example, Tiburcio et al.^72^ only reported adherence for the nine participants at the second follow-up, although there were twenty-three participants at baseline and thirteen at post-intervention follow-up. This introduces a gap in the available data and future studies should be more mindful of which subset of participants they are reporting their process outcomes on.

There were also major differences between how studies administered modules. For example, most computer-based training programs for cognitive–behavioural therapy studies intended for the participant to complete one module every week. On the other hand, some studies, like the one conducted by,^74^ provided their seven modules at much longer intervals over 18 months after discharge from residential drug and alcohol treatment (e.g. the first module on the day of discharge, the second module between day 31 and 60, etc.). The mean number of modules accessed was 1.75 (SD = 2.09;^74^). The effects of this scheduling have not been investigated, but it is possible that the nature of adherence is different for these kinds of longer time intervals. Studies that did not provide modules on a weekly or biweekly regular schedule were excluded from these analyses. Another major limitation when studying adherence in this way is that modules can be very different in terms of the amount of content between interventions. Most interventions provide shorter modules with, that may take around 15–20 min to complete, but some interventions provide fewer comprehensive modules (e.g. see Deady et al.^59^). No study or review to our knowledge has examined the impact of module duration on adherence. In the case of the study by Deady et al.,^59^ the intervention consists of four 1-h modules, each meant to be completed in a separate week over the course of four weeks. The adherence rate for this study, as measured by the mean proportion of modules completed, was relatively low (0.375), which may be explained by the length of the modules.

### Meta-regression

Four predictors were tested for their effects on adherence: guidance, blended treatment, intervention duration and recruitment strategy. Guidance, blended treatment, and recruitment strategy were statistically significant predictors of adherence, while recruitment strategy accounted for the largest amount of heterogeneity found in the meta-analysis (*R^2^* = 66.6%).

Programs that offered blended treatment as compared to standalone e-health interventions, had higher adherence rates. One way to explain this finding is that blended treatment might address a wider range of needs and, thus, may result in more individuals having more of their needs met. In addition, blended treatment programs may inherently provide more human support and face-to-face contact. Investigations into the confounding effects of human contact on predictors of adherence such as blended treatment models and guidance are necessary in future research on adherence. The amount of heterogeneity accounted for was 15.2% (p = 0.02), so this was not as strong a predictor as the other two significant variables.

Recruitment strategies were roughly categorized into local and global strategies. Global strategies are centered around recruitment and advertising through online platforms and social media channels. Local recruitment strategies are conventional in SU research and include recruitment directly in or through clinics and treatment centers, as well as advertisements in local media (such as radio or newspapers). Local recruitment may also suggest that participants were present at the respective clinic or treatment center for attending their sessions or completing their modules and this may have added another layer of human support and accountability, although one review did not find significant differences in adherence between home-based and clinic-based programs.^75^ Although the recruitment variable had a significant and strong effect on predicting adherence, further investigations are necessary to unpack the concept and identify the underlying factors that may be primary in producing these findings. The two recruitment categories may inherently carry an array of factors such as enhanced accountability through additional human support or contact.

Consistent with previous findings and hypotheses in related research, guidance was a significant predictor of adherence (22.19% of heterogeneity accounted for; p = 0.006). Some previous studies show that implementing human support, for example in the form of a clinician or non-clinician, may improve adherence outcomes.^11,76,77^ Although interventions that provide additional guidance or support may be able to increase adherence, it is important to note the drawbacks of this model as it may not be as scalable as self-help models without guidance or support.^76^ A meta-analysis of e-health interventions for cannabis use compared the effects of guided and unguided interventions and did not find a difference.^31^ Further research is necessary to understand the contexts in which different types of guidance may lead to increased adherence and treatment effects. Human support has been thought to enhance adherence through accountability by accompanying the participants through the intervention.^33,77^ To allow a deeper dive into this topic, it is necessary for studies to report the details of the guidance provided, such as the type of training provided to the guides, their expertise, and their amount of contact with the participants.

The duration of the intervention (in weeks) was not a significant predictor of adherence in the present meta-regression. This result may not come as a surprise as a previous review was not able to establish a correlation between adherence and the duration of the intervention.^24^ In addition, there was not a large amount of variance in the durations of the studies as most studies had a duration of around 8 weeks. It is important to note that there are also a multitude of factors that may be at play, as the duration of the intervention (in weeks) by itself does not account for the amount of content provided or the levels of contact established with the patient.

There may be many additional predictors of adherence to e-health interventions for SU that were not subject to our analyses, but that may be interesting for future research and development. These include but are not limited to covariates such as tailoring the intervention, reminders, treatment credibility, and participant characteristics. Tailoring interventions to reflect an individual's symptoms, demographics, interests, and technology usage may be a solution to increasing engagement in self-help models and to increasing the reach of effective, customized interventions. ^78^ In particular, in terms of participant characteristics, more studies need to use individual patient data to understand the different target populations’ needs and their responses to specific interventions. Factors that have been highlighted as important in their effect on adherence are age, gender/sex and the severity of symptoms (or substance use history more broadly.^11,23,79,80^ It has been proposed that generally, self-help interventions are more beneficial for populations with milder symptoms compared to high-risk populations.^81^

### Limitations

In this study, many studies had to be excluded due to a variety of reasons, many related to whether or how adherence was reported. We also excluded interventions that were only provided in a single session, which excluded brief and personalized normalized feedback interventions among others. This set of selection criteria may have introduced bias. Notably, the program that frequently fulfilled these inclusion criteria is computer-based training for cognitive behavioural therapy (CBT4CBT), a widely recognized and effective e-health intervention for substance use (SU).^55^ Therefore, the present results need to be interpreted with care as they do not reflect the needs of all populations and reviews and studies should specifically examine adherence in specific sub-populations (e.g. young adults, opioid users, etc.), to better understand what factors may predict adherence in those contexts.

The two major limitations of this study are the heterogeneity in adherence measures reported and the availability of data. Different types of measures may be used to report adherence, and many e-health studies do not report any adherence data. Furthermore, for the adherence measures used in the present review, it may not always be clearly defined what completion means, and the structure of the modules may be different. In summary, a lot of the information needed to carry out this review and the analyses were based on brief descriptions of authors in the studies, and thus limiting in terms of the level of details provided. In particular, in terms of predictors of adherence, such as guidance, this has been mentioned by other reviews as a limitation in trying to get a complete picture of how guidance is provided in a study.^76,81^

Another limitation is the exclusion of recently published articles from 2021 to 2023. The evaluation of articles from this time period could be a subject for future investigation. Furthermore, the lack of a published protocol in this review is a limitation, as it raises concerns about research transparency, reproducibility, and potential bias. Future reviews should prioritize publishing protocols to enhance the rigor and reliability of their research.

## Conclusion

Despite many challenges and limitations, the findings of the present review can help generate future hypotheses for investigating adherence, not only in e-health for SU but also in the mental health field. The results suggest that there is a lot of room for improvement in adherence to e-health interventions for SU and that three factors may play an essential role in influencing adherence: guidance, recruitment and the integration of blended treatment. We recommend that future studies investigate these three factors in more detail, first to support the findings and second to uncover the underlying mechanisms that may lead to these results. It would also be interesting to continue and study the impacts of different types of human contact on adherence and how they may correlate with other predictive factors.

The conclusions of this review should be used to improve research designs and report in future trials to support research related to adherence, which may ultimately have a significant influence on the development and effectiveness of new or updated interventions. Making these e-health interventions more engaging is key to ensuring their impact and effectiveness for as many people as possible.

## Supplemental Material

sj-docx-1-dhj-10.1177_20552076231203876 - Supplemental material for Adherence to e-health interventions for substance use and the factors influencing it: Systematic Review, meta-analysis, and meta-regression Click here for additional data file. Supplemental material, sj-docx-1-dhj-10.1177_20552076231203876 for Adherence to e-health interventions for substance use and the factors influencing it: Systematic Review, meta-analysis, and meta-regression by Farhud Shams, Andy M.Y. Tai, Jane Kim, Marisha Boyd, Maximilian Meyer, Alireza Kazemi and Reinhard Michael Krausz in DIGITAL HEALTH
