# Supplementary material for: Adherence to e-health interventions for substance use and the factors influencing it: Systematic Review, meta-analysis, and meta-regression
Source: Digit Health. 2023 Sep 28;9:20552076231203876. doi: 10.1177/20552076231203876 (PMC10540609; doi:10.1177/20552076231203876)
Supplement: sj-docx-1-dhj-10.1177_20552076231203876 - Supplemental material for Adherence to e-health interventions for substance use and the factors influencing it: Systematic Review, meta-analysis, and meta-regression [file sj-docx-1-dhj-10.1177_20552076231203876.docx]

Search Query

( (substance us* ORsubstance addict* ORsubstance depend* ORsubstance misuse ORdrug addict* OR drugdepend* OR alcohol us*)AND (online OR internet-based OR web-based ORe-health OR e-mentalhealth OR emental healthOR computer*) AND(adher* OR engag* ORcompliance) AND(Intervention ORprevention OR treatment)) NOT meta-analysis NOTsystematic review

Table 1. Summary of included studies.

| Study ID | Country | Interventions | Study Design | Target Substance |
| --- | --- | --- | --- | --- |
| Acosta 2017 | United States | Thinking Forward | RCT | General SU |
| Batterham 2018 | Australia | A: FitMindKit - tailored | RCT | General SU |
|  |  | B: FitMindKit - static |  |  |
| Brooks 2010 | United States | TES | Feasibility | Cocaine use |
| Budney 2011 | United States | Computerized MET/CBT/CM intervention | Non-randomized trial | Cannabis use |
| Campbell 2014 | United States | TES | RCT | General SU |
| Carroll 2008 | United States | CBT4CBT | RCT | General SU |
| Carroll 2014 | United States | CBT4CBT | RCT | Cocaine use |
| Carroll 2018 | United States | A: CBT4CBT + Galantamine | RCT | Cocaine use |
|  |  | B: CBT4CBT + Placebo |  |  |
| Chaple 2014 | United States | TES | Randomized open-label trial | General SU |
| Deady 2016 | Australia | DEAL Project (based on SHADE) | RCT | Alcohol use |
| Study ID | Country | Interventions | Study Design | Target Substance |
| Johansson 2017 | Sweden | eChange | Pre-post observational | Alcohol use |
| Johansson 2020 | Sweden | ICBT | RCT | Alcohol use |
| Kay-Lambkin 2009 | Australia | SHADE | RCT | Alcohol/cannabis use |
| Kiluk 2016 | United States | A: CBT4CBT + TAU | RCT | Alcohol use |
|  |  | B: CBT4CBT + brief clinical monitoring |  |  |
| Kiluk 2018 | United States | CBT4CBT plus monitoring | RCT | General SU |
| Marsch 2014 | United States | TES | RCT | Opioid use |
| Paris 2018 | United States | CBT4CBT-Spanish | RCT | General SU |
| Schaub 2012 | Switzerland | SnowControl | RCT | Cocaine use |
| Schaub 2015 | Switzerland | A: CanReduce + chat counselling | RCT | Cannabis use |
|  |  | B: CanReduce |  |  |

| Study ID | Country | Interventions | Study Design | Target Substance |
| --- | --- | --- | --- | --- |
|  |  |  |  |  |
| Schaub 2019 | Switzerland | A: SnowControl + chat counselling | RCT | Stimulant use |
|  |  | B: SnowControl |  |  |
| Shi 2019 | Australia | CBT4CBT for office-based buprenorphine | Pilot RCT | Opioid use |
| Sundstrom 2020 | Sweden | A: ePlus (high intensity intervention) | RCT | Alcohol use |
|  |  | B: eChange (low Intensity Intervention) |  |  |
| Takano 2020 | Japan | e-SMARPP | Pilot RCT | General SU |
| Tiburcio 2018 | Mexico | PAADD | Pilot randomized open-label trial | General SU |

*Note.* TES = Therapeutic Education System; MET = Motivational Enhancement Therapy; CBT = Cognitive-Behavioural Therapy; CM = Contingency Management; PAADD = Programa de Ayuda para Abuso de Drogas y Depresión; e-SMARPP = e-learning Serigaya Methamphetamine Relapse Prevention Program; TAU = treatment as usual; RCT = randomized controlled trial; SU = substance use.
